# Applying microbial biostimulants and drought-tolerant genotypes to enhance barley growth and yield under drought stress

**DOI:** 10.3389/fpls.2024.1494987

**Published:** 2025-01-07

**Authors:** Mohamed Ferioun, Ilham Zouitane, Said Bouhraoua, Yasmine Elouattassi, Douae Belahcen, Abdellatif Errabbani, Said Louahlia, Riyaz Sayyed, Naïma El Ghachtouli

**Affiliations:** ^1^ Natural Resources and Environmental Laboratory, Taza Polydisciplinary Faculty, Sidi Mohamed Ben Abdellah University, Fez, Morocco; ^2^ Microbial Biotechnology and Bioactive Molecules Laboratory, Sciences and Technology Faculty, Sidi Mohamed Ben Abdellah University, Fez, Morocco; ^3^ Department of Biological Science and Chemistry, College of Arts and Science, University of Nizwa, Nizwa, Oman

**Keywords:** Bio-inoculant; drought stress, genotypes, *Hordeum vulgare*, microbial biostimulants, PGPR

## Abstract

With climate change, the frequency of regions experiencing water scarcity is increasing annually, posing a significant challenge to crop yield. Barley, a staple crop consumed and cultivated globally, is particularly susceptible to the detrimental effects of drought stress, leading to reduced yield production. Water scarcity adversely affects multiple aspects of barley growth, including seed germination, biomass production, shoot and root characteristics, water and osmotic status, photosynthesis, and induces oxidative stress, resulting in considerable losses in grain yield and its components. In this context, the present review aims to underscore the importance of selecting drought-tolerant barley genotypes and utilizing bio-inoculants constructed from beneficial microorganisms as an agroecological approach to enhance barley growth and production resilience under varying environmental conditions. Selecting barley genotypes with robust physiological and agronomic tolerance can mitigate losses under diverse environmental conditions. Plant Growth Promoting Rhizobacteria (PGPR) play a crucial role in promoting plant growth through nutrient solubilization, nitrogen fixation, phytohormone production, exopolysaccharide secretion, enzyme activity enhancement, and many other mechanisms. Applying drought-tolerant genotypes with bio-inoculants containing PGPR, improves barley's drought tolerance thereby minimizing losses caused by water scarcity.

## Graphical Abstract

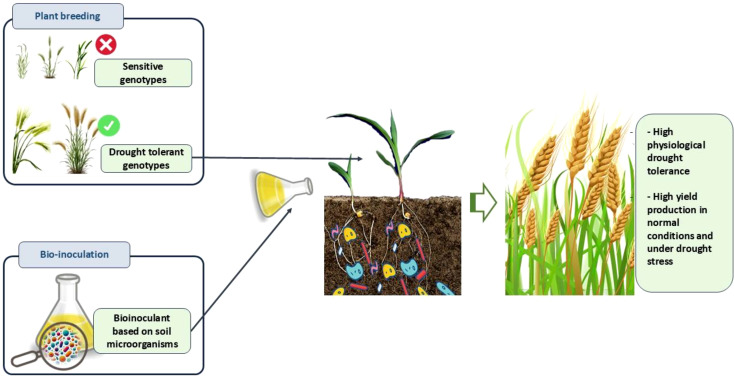

## 1 Introduction

Barley (*Hordeum vulgare* L.) comes in fourth place among most cereals cultivated around the world. Historically, barley is among the first domesticated crops (Geng et al., 2021). Now, it is widely used in food, animal feed, brewing, distillation, forage, and many other industrial uses (Bouhraoua et al., 2024). Ensuring barley supply is becoming increasingly challenging due to the continuous increase in the global human population, especially considering that the majority of individuals depend on agriculture for their livelihoods (Castaneda et al., 2016). Indeed, the risks associated with abiotic stresses such as drought, salinity, and heat are escalating annually due to climate change.

Drought is among the major stresses that affect barley crops and can cause more than 50% of yield losses (Kebede et al., 2020). Several studies showed ridiculous changes in barley plants subjected to drought stress when compared to the unstressed ones. Drought affects root and shoot length and weight (Abou-Elwafa, 2016), reduces relative water content (Hasanuzzaman et al., 2019), affects membrane steadiness (Ferioun et al., 2024), alters photosynthesis efficiency (Ghotbi-Ravandi et al., 2014), induces some metabolites accumulation, stimulates reactive oxygen species accumulation (Sallam et al., 2019) and many other physiological changes, which impacts the grain yield (Ferioun et al., 2024). Hence, increasing barley production by expanding land areas for cultivation is not a straightforward solution. Furthermore, relying solely on chemical fertilizers is not an agroecological solution.

Selecting high-performing genotypes is among the solutions that many studies have endeavoured to pursue with the aim of minimizing crop losses attributable to water scarcity. It is challenging to select the most high-yielding genotypes due to significant genotype x environment interactions (Boussakouran et al., 2021). Therefore, testing various genotypes in different environments is widely recommended to identify ideal genotypes that shown high yield production and are less affected by stresses (Boussakouran et al., 2021; Bouhraoua et al., 2023).

Investigating alternative strategies to increase barley output while reducing the negative environmental effects associated with traditional agricultural techniques has gained traction in recent years. A bio-inoculant based on Plant Growth Promoting Rhizobacteria (PGPR), mycorrhizae (Vafa et al., 2021; Jabborova et al., 2022; Vafa et al., 2024), and/or algae is considered as an agroecological solution that offers numerous benefits to various crops in both non-stressful and stressful conditions Ahluwalia et al., 2021; Al-Turki et al., 2023). The selection of microorganisms used for bio-inoculant production is mostly based on plant growth promoting traits such as nutrients solubilization, phytohormones production, nitrogen fixation, exopolysaccharides secretion, and enzymes activities (Etesami and Maheshwari, 2018; Kumar et al., 2019).

In barley growth, bio-inoculants have demonstrated a significant improvement in various environmental conditions. Barley plants inoculated with bio-inoculants, when compared to uninoculated ones, exhibited higher leaf relative water content, increased biomass production, improved grain quality and quantity, and lower sensitivity indices (Baris et al., 2014; Jodeh et al., 2015; Slimani et al., 2023a). Furthermore, the positive effects of bio-inoculants extend to improving soil health in the rhizosphere (Tirry et al., 2023). The utilization of bio-inoculants as fertilizers in the field is continuously progressing, although it remains limited compared to chemical fertilization practices (Bittencourt et al., 2023). In light of the numerous disadvantages associated with chemical fertilizers, bio-inoculants appear to offer a sustainable and agroecological solution for enhancing barley resilience to drought.

The present study aimed to investigate bio-inoculant advancements and breeding techniques to enhance barley tolerance to drought conditions. We also explored the potential synergistic approach of combining robust barley genotypes with biofertilizers for improved crop production in barley fields.

## 2 Drought stress in the context of global climate change

In recent decades, there has been growing concern about worsening global weather conditions and its impact on agriculture. The persistent build-up of greenhouse gases (GHGs) in the Earth's atmosphere is forecasted to intensify soon. This escalation is expected to elevate the average global temperature, alter precipitation patterns, and amplify the occurrence and intensity of extreme weather events. Consequently, this leads to the phenomenon known as climate change, marked by reduced rainfall, prolonged droughts, intense winds, or flooding that can damage crops and lead to losses after harvesting (Toulotte et al., 2022; Mutengwa et al., 2023). One of the biggest challenges is the increase in drought and water scarcity (Vila-Traver et al., 2021; Bogati and Walczak, 2022). The future prediction indicates that climate change will result in heightened occurrences of droughts in most parts of the world. The climate forecasts indicate a potential rise in average temperatures ranging between 0.5° to 5.6°C, varying according to different scenarios. Moreover, these projections suggest a continual decrease in rainfall between April to September, estimating a reduction of approximately 4% for every degree Celsius of global warming (Cramer et al., 2020). The affected area is anticipated to surge from 15.4% to 44% by the year 2100 and Africa is identified as the most vulnerable region (Idris et al., 2022). Irregular and inadequate rainfall has serious and detrimental effects on crop production and crop quality, especially in tropical regions where most of the world’s water is used for agriculture (Reynolds and Ortiz, 2010; Kebede et al., 2020). The impact of drought varies across time and space, from the local level to the regional level and then to the global level (Mishra et al., 2015). According to Cavicchioli et al. (2019); Toulotte et al. (2022), rising temperatures and drought significantly impact crop growth. Forecasts suggest that in areas affected by drought, the yields of major crops could decrease by over 50% by 2050 and nearly 90% by 2100 (Li et al., 2009). Multiple separate studies have highlighted the impact of elevated temperatures and water stress on agricultural yields. For instance, in Canada, severe events that occurred in 2001 and 2002, along with droughts and floods in 2010 and 2011, significantly devastated crop yields, resulting in reductions of up to 50% (Yang et al., 2020). Additionally, from 1980 to 2016, substantial disasters in the United States, each surpassing a billion dollars annually, underscore the significant agricultural losses exceeding $220 billion stemming from the combined effects of drought and heat (Lamaoui et al., 2018). Among various abiotic stresses, drought is considered as the most detrimental since it poses a major challenge to sustainable food production, as it can reduce the potential yield of different crops by up to 70% (Gosal et al., 2009). Drought can exert a significant toll on both human well-being and agricultural activities. They are estimated to impact around 55 million individuals annually worldwide, representing a major menace to livestock and crops on a global scale (Francini and Sebastiani, 2019; Elakhdar et al., 2022). Molénat et al. (2023) assert that climate change amplifies the challenges confronting Mediterranean agriculture, particularly in maintaining or improving production levels to meet the growing demands of an expanding population. The Mediterranean diet, comprising representative crops such as olives, grapes, fruits, cereals, and legumes (used as an alternative to animal protein), enjoys global recognition (Cramer et al., 2020). The agricultural sector in the Mediterranean region employs a significant workforce and holds economic importance. For example, in the Maghreb, agriculture engages 13–20% of the workforce and contributes 10–20% to the region's gross domestic product (Cramer et al., 2020). In developing nations, the impact of heat and drought is of paramount significance. In Morocco, where agriculture plays a substantial role in the economy, involving around 40% of the workforce, drought significantly reduces crop productivity. This directly affects farmers' livelihoods and has subsequent repercussions on the overall economy. Reports from West Africa indicate crop failures due to drought, leading to a 25% decrease in per capita food production (Gbegbelegbe et al., 2024). In the Mediterranean region, agriculture is the main user of water. For example, in countries of the Eastern Mediterranean, crop irrigation accounts for up to 79% of total water withdrawals (Hellal et al., 2019). This challenge coincides with the need to protect essential natural resources like water, soil, and biodiversity, which remain susceptible to both climate-related dangers and anthropogenic activities. The effective management of the limited water supply in a warmer and drier climate stands out as one of the most critical concerns confronting Mediterranean agriculture. Climate projections and crop modeling indicate that water scarcity and heat waves significantly restrict and will continue to limit crop production (Francini and Sebastiani, 2019; Molénat et al., 2023). Consequently, the viability of the Mediterranean food system hinges on the ability to sustain Mediterranean agriculture amidst water scarcity.

## 3 Barley and water stress

Barley (*Hordeum vulgare* L.) is the 13^th^ most produced crop in the world and 4^th^ in harvested area, with 1570000 Tons and 51.6 million ha, respectively, it stands as a highly important cereal crop cultivated across regions spanning Europe, the Middle East, North and South Africa, as well as Asia (Elakhdar et al., 2022; Talaat, 2023). In global production, Europe holds a share of 60% with 46% of the total global area dedicated to barley cultivation (Martínez-López et al., 2022). Spain leads in barley cultivation, producing approximately 11000 Tons across 2.75 million hectares, of which 0.36 million hectares are under irrigation (Martínez-López et al., 2022). Barley is a versatile crop with substantial economic impact, serving as a staple food for both humans and animals, as well as in industrial sectors such as brewing (Elakhdar et al., 2022; Toulotte et al., 2022). Drought is characterized as an extended duration of insufficient rainfall, leading to the depletion of soil moisture through transpiration or evaporation, thereby rendering it insufficient to meet the demands of crops (Lamaoui et al., 2018; Francini and Sebastiani, 2019; Toulotte et al., 2022). Several studies conducted globally have demonstrated that water shortage triggered multiple modifications in barley (Hasanuzzaman et al., 2019; Tabatabaei and Ansari, 2020; Talaat, 2023). Drought has been shown to reduce grain yield in barley, by 49–87% (Samarah, 2005; Kebede et al., 2020). Under such circumstances, it becomes increasingly important for farmers and agronomists to focus on enhancing barley's resilience to drought stress, all the while striving to maintain or even improve productivity in challenging climatic conditions.

## 4 Effect of drought stress on crucial barley plant processes

### 4.1 Seed germination

Seed germination is a crucial process for the growth, development, and successful establishment of a plant. It begins with the uptake of water, which activates the essential metabolic processes in the seed. This water uptake is also necessary for the activation of hydrolytic enzymes, which are responsible for the degradation of starch, solubilization, and transport of carbohydrates. However, the scarcity of soil moisture can significantly impact the successful germination and establishment of plants (Wijewardana et al., 2019). This is because of the reduced water uptake rate through the seed coat under stress conditions that can lead to a decrease in the percentage of seed germination (Reza Yousefi et al., 2020). Additionally, germinated seedlings in a moisture-deficit environment show a reduction in both seedling vigor and germination index (Sazegari et al., 2020; Tang et al., 2023). The average time required for seed germination is also extended under soil moisture deficit stress (Queiroz et al., 2019). Furthermore, the fresh weight as well as the dry weight of germinated seedlings decline in a moisture-deficit environment. This reduction in seed hydration can slow down the process of hydrolysing stored carbohydrate material, leading to a lesser and slower supply of hydrolysed material to the embryo axis. This, in turn, can affect the emergence of the radicle from the seed coat, leading to a delay in seed emergence and a reduction in seedling vigour (Channaoui et al., 2019).

### 4.2 Water & nutrients uptake and transport

In times of soil water deficit, the uptake capacity of roots becomes limited due to the reduction in the water potential gradient between the soil and the plant. This is because the root hydraulic conductivity decreases under drought conditions, which in turn limits the water uptake capacity of roots from the soil (Zhu et al., 2024). The expression of genes coding for aquaporin, which controls the root hydraulic conductivity, is generally downregulated under moisture deficit conditions. This downregulation ultimately lowers the root water uptake capacity (Mukarram et al., 2021). Moreover, under stress, the continuous loss of transpiration and lower soil moisture lead to an increase in xylem cavitation. This increase gradually reduces the hydraulic conductance in plants and blocks the movement of water within the plant. This further exacerbates the water stress conditions (Zhu et al., 2024).

In plants, nutrient uptake is a complex process that involves the absorption of nutrients from the soil through water. Water serves as a medium for nutrients to move within the soil matrix and from the soil to the plants. However, soil moisture deficit conditions can significantly decrease the nutrient uptake from the soil. This reduction in nutrient uptake may be attributed to a decrease in the nutrient supply through mineralization, as well as a reduction in the diffusion and mass flow of nutrients in the soil (Duman, 2006; Jorenush and Rajabi, 2015). The kinetics of nutrient uptake by the roots are also affected, reducing the rate of nutrient uptake under drought stress. This is further compounded by the reduced translocation of nutrients from the root to the shoot, which contributes to a reduction in the nutrient status of different plant parts (El-Denary and El-Shawy, 2014). In addition, the microbial growth in the rhizosphere is also affected under deficit soil moisture conditions, ultimately affecting the nutrient uptake by the roots (Jalal et al., 2014). To cope with drought, plants upregulate the expression of different types of transporter proteins to balance the nutrient uptake under stress conditions. Genes encoding for potassium transporter proteins are also upregulated under drought stress (Maleki Farahani et al., 2010; Abdel-Ghani et al., 2015). Bista et al. (2018) reported that the reductions in P uptake under drought conditions were correlated with decreases in P-uptake protein concentration and activity. However, reductions in nitrogen (N) uptake were only inversely related to N-uptake protein levels. Due to greater decreases in total protein per gram, the concentration of nutrient-uptake proteins per gram significantly declined, despite increases in total protein per gram. Therefore, it is plausible that decreases in the concentration of root nutrient-uptake proteins in both drought-tolerant and sensitive species contributed to at least some of the drought-related declines in nutrient concentration, particularly in %P. In conclusion, while drought stress can negatively affect nutrient uptake from the soil, plants also develop strategies to challenge stressful conditions through the synthesis of a greater number of transport proteins to fulfill their nutrient requirements.

### 4.3 Physiological responses

#### 4.3.1 Growth

Severe stress conditions significantly impact various aspects of germination, seed vigour, and seedling characteristics in different cereals genotypes (Dhanda et al., 2004). This stress, characterized by rough conditions, leads to delays, reductions, or inhibitions in the germination process and the overall vigour of cereal seeds (Ghanifathi et al., 2011; Tabatabaei, 2013). Additionally, it influences the Germination Stress Tolerance Index (GSTI) (Moayedi et al., 2009; Barati et al., 2015). Under water-deficit conditions, the seed vigour index emerges as the most susceptible trait, followed by root and shoot length, and germination percentage (Moayedi et al., 2009). The repercussions of drought stress extend to the reduction of cell membrane stability, changes in relative water content (Geravandi et al., 2011), early maturity, diminished leaf area (Tabatabaei and Ansari, 2020), decreased dry weight, and alterations in the root–shoot ratio (Shi et al., 2014). Furthermore, the overall response of the whole plant, including the transpiration rate, to elevated atmospheric vapor pressure deficit is linked to drought tolerance in cereals (Schoppach et al., 2017).

#### 4.3.2 Root and shoot characteristics

**Figure 1:**
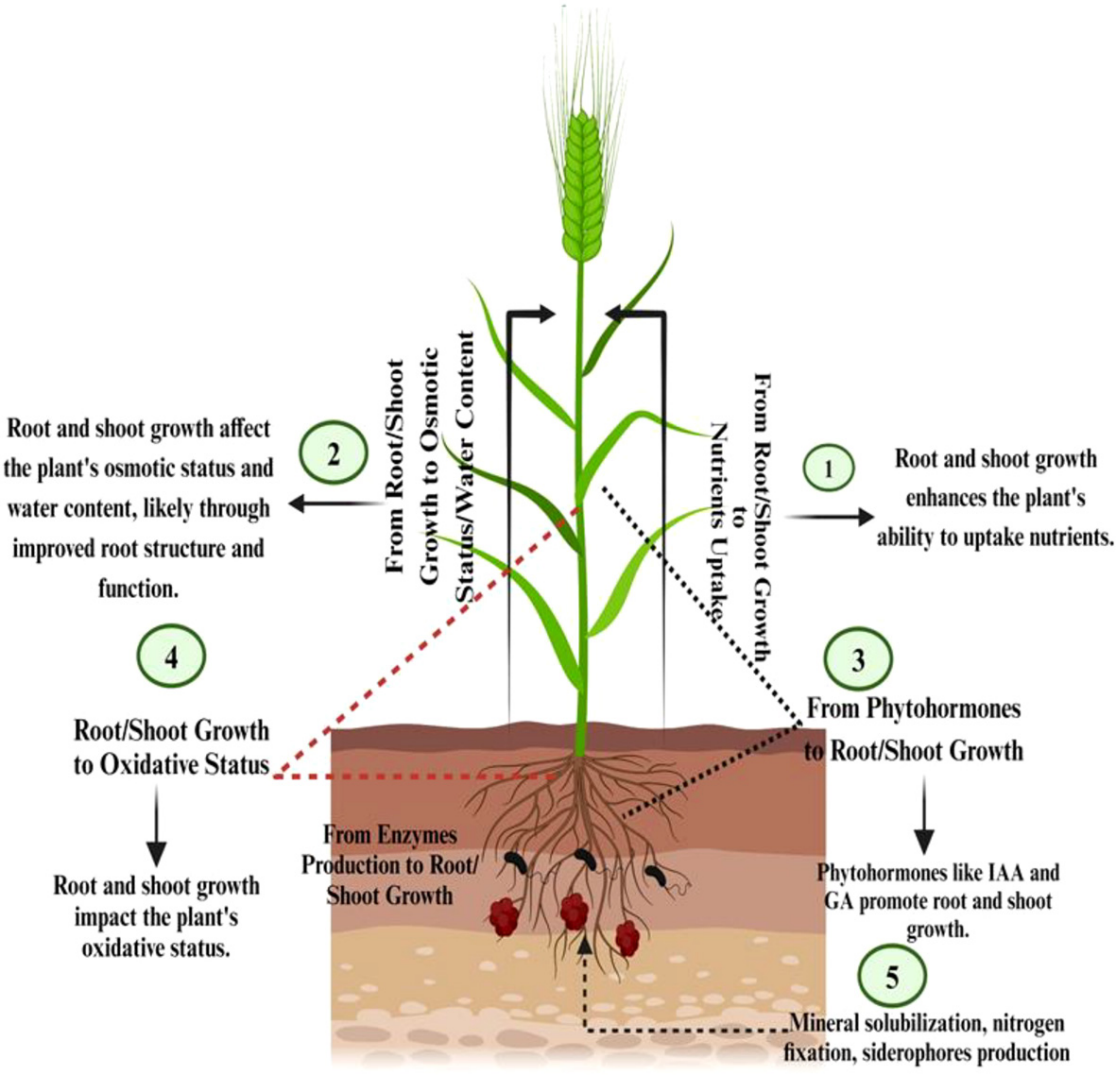
Mechanisms of barley drought tolerance: interaction between root/shoot growth, nutrient uptake, and physiological processes.

Morphological characteristics of barley affected by water deficiency encompass various leaf features, including shape, area, expansion, size, waxiness, pubescence, senescence, and cuticle tolerance. Additionally, root traits such as length, density, fresh and dry weight are influenced by water deficit (Hasanuzzaman et al., 2020). Assessing leaf water potential proves to be an effective and dependable method for gauging plants' response to water scarcity. Under water-limiting conditions, the gradual reduction in electron transport of photosystem II occurs, leading to an increase in non-photochemical quenching capacity. Consequently, this results in a decline in cereal leaf relative moisture content (Jouyban et al., 2015). Drought conditions contribute to a decrease in leaf water potential in barley plant, attributed to solute accumulation. However, there may be genotypic variations in the response to water potential, both under well-watered and drought conditions (Istanbuli et al., 2020). Leaf water potential also impacts various gas exchange characteristics, including stomatal conductance, net-photosynthetic rate, and transpiration (Hasanuzzaman et al., 2019) (**Figure 1**).

#### 4.3.3 Water status

Relative water content (RWC) serves as a crucial indicator of water status in barley experiencing water deficit, in contrast to leaf water potential (Sallam et al., 2019). This parameter has proven instrumental in selecting drought-tolerant barley cultivars (Teulat et al., 2003). Notably, in cereals, the negative impact of drought intensifies when applied later in plant growth (beyond 6 weeks post-emergence), leading to more detrimental effects on water relations, nutrient uptake, growth, and yield, compared to early drought imposition (after 3 weeks of seedling emergence) (Ghotbi-Ravandi et al., 2014). Chlorophyll content, membrane stability, and RWC of barley cultivars during the flowering stage are significantly reduced under drought stress (Hebbache et al., 2021). A study found that drought stress caused a substantial 43% reduction in relative water content (from 88% to 45%) in four genotypes of wheat plant (Siddiqui et al., 2021). This reduction in RWC leads to stomatal closure, subsequently diminishing the rate of photosynthesis. Water scarcity hampers osmotic regulation, but alternating between drying and re-watering induces osmotic regulation, improving the plant's water use efficiency during drought conditions. A tolerance strategy to water deficit involves maintaining high relative moisture content, attributed to increased osmotic regulation (Alghabari and Ihsan, 2018). Drought-tolerant genotypes demonstrate the ability to sustain high turgor potential and relative water content, indicating minimal impact on their protoplasmic structure compared to sensitive genotypes. This correlation between RWC and photosynthetic rate underscores the importance of maintaining leaf turgor as an adaptive mechanism crucial for stomatal regulation and photosynthetic activities under water-deficit conditions (Elakhdar et al., 2022).

#### 4.3.4 Osmotic status

Osmoregulation is the active process of lowering osmotic potential in plant cells through the accumulation of osmotically active compounds (Sallam et al., 2019). It is an indispensable and effective component of plant resistance to drought, in particular because it maintains the turgor pressure associated with a large number of metabolic and physiological processes for which the presence of cell turgor is crucial (Zivcak et al., 2016).

Plants employ various strategies to maintain their water balance when faced with drought. Three methods of osmotic regulation exist, intracellular water reduction, wherein plants decrease the amount of water inside their cells to reduce osmotic potential and conserve the free energy of water, cell volume reduction, where cells reduce in size to maintain osmotic pressure, enabling plants to better resist water stress, and increase in cell content, whereby plants elevate the concentration of solutes inside their cells (Sallam et al., 2019; Ahluwalia et al., 2021). This lowers osmotic potential and facilitates water uptake, even when external water potential is low (Wang et al., 2000). While these mechanisms coexist in plants, not all possess the same capacity for osmotic regulation (Osakabe et al., 2014) (**Figure 1**).

Ultimately, osmotic regulation is necessary to maintain the required turgor pressure for cell growth, even in water-stressed periods (Sanders and Arndt, 2012; Osakabe et al., 2014). Osmotic regulation is an important mechanism that helps maintain stomatal conductance and turgor pressure in plants when there is a moderate water deficit. This mechanism is crucial for keeping a high concentration of CO_2_ in the intercellular space of the mesophyll, which prevents or reduces the inhibition of photosynthesis (Osakabe et al., 2014). Osmotic regulation also plays a vital role in protecting various cellular processes like cell growth, stomatal opening, and photosynthesis under environmental stress (Sallam et al., 2019).

Certain compounds, such as amino acids, amino acid derivatives, trehalose, fructan, and mannitol, act as osmotic regulators and help control the osmotic pressure in the cytoplasm (Wang et al., 2000). These substances have low molecular weight, high solubility, and low toxicity, making them effective in maintaining normal osmotic pressure and preserving the activity of proteins and the integrity of the cell membrane structure (Sanders and Arndt, 2012; Osakabe et al., 2014; Sallam et al., 2019). Currently, a large number of studies are focusing on osmotic regulating substances such as proline, soluble sugars, glycine betaine, among others. Research has shown that proline accumulation represents a protective strategy adopted by plants to cope with drought stress (Yang et al., 2021).

#### 4.3.5 Photosynthesis efficiency

One essential metabolic function greatly impacted by drought is photosynthesis. In response to water scarcity, roots produce abscisic acid (ABA), causing stomatal closure, which reduces gas exchange and inhibits photosynthesis (Sallam et al., 2019; Ahluwalia et al., 2021; Qiao et al., 2024). Drought affects various subcellular regions and organelles, slowing overall plant growth, preventing seed germination, and hindering cell elongation (Fadiji et al., 2022). Drought-stressed plants are shorter and have fewer leaves, which limits their ability to absorb photosynthetically active radiation (PAR) and consequently restricts both photosynthesis and yield (Fadiji et al., 2022). Additionally, the lack of moisture in the soil increases salt concentration, which lowers the water potential of plant cells and impedes growth (Mondini and Pagnotta, 2015; Mahboob et al., 2023; Nazim et al., 2024). As a result, drought disrupts the mass movement of essential water-soluble minerals, such as calcium, magnesium, and nitrate, all vital for plant development (ElSayed et al., 2022; Qiao et al., 2024. Ahluwalia et al., 2021). These changes highlight the critical importance of water availability for overall plant functioning and growth (Zargar et al., 2017; Fadiji et al., 2022; Benito-Verdugo et al., 2023; Iqbal et al., 2023; Kumar et al., 2024; Qiao et al., 2024).

Furthermore, stomatal closure decreases stomatal conductance, leading to increased photorespiration and further restricting photosynthesis (Fadiji et al., 2022). This inhibition likely results in plants absorbing more light energy than they can effectively utilize for carbon fixation (Jedmowski et al., 2013; Ghadirnezhad Shiade et al., 2023; Luqman et al., 2023). Key components limiting photosynthesis include reduced CO_2_ diffusion due to early stomatal closure, decreased activity of photosynthetic enzymes, biochemical changes related to triose-phosphate formation, and reduced photochemical efficiency of photosystem II (Ghadirnezhad Shiade et al., 2023; Li and Wang, 2023; Qiao et al., 2024). The imbalance between light capture and its utilization leads to the accumulation of reactive oxygen species (ROS) in the chloroplast and, subsequently, the disorganization of thylakoid membranes, decrease in Rubisco activity, loss of chloroplast membranes, degradation of chloroplast structure and photosynthetic apparatus, chlorophyll photo-oxidation, destruction of chlorophyll substrate, inhibition of chlorophyll biosynthesis, and the increase of chlorophyllase activity (Kirkby et al., 2023; Rodrigues et al., 2024; Sharma et al., 2024).

While reduced stomatal conductance minimizes water loss through leaves (Li and Wang, 2023; Qiao et al., 2024), it simultaneously limits photosynthesis by decreasing intercellular CO_2_ levels (Benito-Verdugo et al., 2023; Ghadirnezhad Shiade et al., 2023; Jahan et al., 2023). Consequently, these interconnected disturbances lead to reduced water use efficiency (WUE) in plants (Sallam et al., 2019; Kirkby et al., 2023; Li and Wang, 2023; Mahboob et al., 2023; Kumar et al., 2024; Qiao et al., 2024).

#### 4.3.6 Oxidative status

The production of reactive oxygen species (ROS) in response to stress represents the primary reaction of plant cells to various environmental stresses. These ROS cause cellular damage by altering proteins, inactivating enzymes, disrupting gene expression and altering membranes (Amirjani and Mahdiyeh, 2013). In parallel, changes in ROS generation act as signals, influencing gene transcription and thus contributing to plant adaptation to abiotic stresses (Rajasekar et al., 2015). The key members of the ROS family, such as singlet oxygen (O), superoxide radicals (O_2_), hydrogen peroxide (H_2_O_2_) and hydroxyl radical (OH), are responsible for oxidative damage in plants (Sallam et al., 2019). Antioxidant enzymes regulate ROS at low levels under normal conditions, ensuring stability (Hasanuzzaman et al., 2020). However, a decrease in antioxidants or an increase in ROS production can upset this balance, resulting in oxidative stress. The latter results in damage to macromolecules and cell membranes, as well as increased lipid peroxidation (Ansari et al., 2019). Drought induces increased ROS production, leading to a rise in malondialdehyde (MDA) levels (Istanbuli et al., 2020). This compound is considered a reliable indicator of oxidative damage, particularly as a marker of membrane lipid peroxidation (Pakzad et al., 2019). Reduced membrane stability thus reflects the level of ROS-induced lipid peroxidation (Murtaza et al., 2016). Low MDA levels are linked to drought stress tolerance in cereals (Zhang et al., 2011).

The main ROS production sites in plants are located in the chloroplast, mitochondria and peroxisomes, while the endoplasmic reticulum, cell membrane, cell wall and apoplast act as secondary production centers (Sallam et al., 2019). To protect themselves against the damaging effects of ROS, plants have developed an effective antioxidant system, consisting of both enzymatic antioxidants, such as superoxide dismutase (SOD), catalase (CAT), ascorbate peroxidase (APX), guaiacol peroxidase (GPX), glutathione reductase (GR), mono dehydro-ascorbate reductase (MDHAR) and dehydro-ascorbate reductase (DHAR), as well as non-enzymatic antioxidants, including ascorbic acid, reduced glutathione (GSH), α-tocopherol, carotenoids, flavonoids and proline. These two antioxidant systems work in tandem to neutralize ROS (Das and Roychoudhury, 2014).

### 4.4 Agronomic traits

Drought stress hurts flowering and grain development processes during the reproductive stage, which reduces spikelet fertility, results in poor grain filling, and reduces grain weight (Thabet et al., 2020). Consequently, compared to non-stressed plants, barley plants in drought conditions frequently display decreased grain production, smaller grain size, and a poorer harvest index (Samarah et al., 2009; Khalili et al., 2016). The impact of drought is not solely determined by its duration but also by the timing of water scarcity (Dietz et al., 2021). When drought occurs during the germination stage, it results in significant losses in plant density (Farooq et al., 2012). Conversely, water shortage during the vegetative stage leads to reductions in biomass production, particularly affecting leaf area, especially the flag leaf (Biswal and Kohli, 2013; Tian et al., 2022), which in turn affects yield production. This relationship is supported by the strong association between leaf area and grain yield (Jabbari et al., 2019). Water scarcity during the flowering stage (anthesis) has a detrimental effect on fertility, leading to a reduction in the number of spikes per plant (Jamieson et al., 1995; Dietz et al., 2021). Post-anthesis drought impacts grain filling by diminishing seed weight, thereby reducing grain yield due to decreased carbohydrate reserves. Consequently, these effects extend to the subsequent generation (Dietz et al., 2021).

Drought stress can also change the protein content, starch composition, and malting properties of barley grains (Mahalingam, 2017), which can have an impact on the food and beverage sectors. Overall, the impact of drought stress on barley agronomic traits highlights the necessity of all-encompassing approaches, such as the creation of drought-resistant cultivars and the application of water-saving agronomic techniques, to improve drought tolerance and reduce yield losses in barley production.

## 5 Methods for mitigation of drought stress in barley plants

### 5.1 Selection of drought-tolerant genotypes

#### 5.1.1 methods adopted for genotypes selection

Drought tolerance might be approached differently, making it very complicated. Successful plant breeding requires effective assays to select the drought-tolerant genotypes (Sallam et al., 2019). In literature, there are many methods for selecting barley drought-tolerant genotypes, each method is based on various traits and can be adopted in different growth phenological stages (**Table 1**). Many screening studies were effected at the germination stage by estimating the germination rate and speed (Barati et al., 2015; Hellal et al., 2018; Adriana, 2020). Drought stress decreased germination percentage and speed, with a higher effect on germination speed than germination percentage (Hellal et al., 2018). Autumn rainfall water deficit can slow down and inhibit germination, which affects yield production (Adriana, 2020). Based on this problematic, breeders think that the selection of barley genotypes that can preserve a high germination rate and speed under water shortage conditions might guarantee good barley plant growth in various environmental conditions (Khafagy et al., 2017).

**Table 1:** Studies performed for selecting barley drought genotypes based on various physiological and agro-morphological traits.

Genotypes	Origin	Stresses	Growth stage	Selection traits	References
Nine Moroccan cultivars	Morocco	Drought stress in pots in greenhouse under controlled conditions	Tillering stage	▪ Biomass production ▪ Physiological traits ▪ Biochemical traits	(Ferioun et al., 2023b)
Twenty-five Hordeum vulgare	Iran	Drought stress in pots in greenhouse under controlled conditions	Full maturity	▪ Drought tolerance indices	(Saed-Moucheshi et al., 2021)
Otis and Baronesse genotypes	United States of America	Water shortage in plastic pots under controlled conditions	Tillering stage	▪ Hormonal profiling ▪ Gas exchange measurements ▪ Metabolites content (Proline, malonaldehyde ▪ differential gene expression	(Harb et al., 2020)
- landrace-derived lines SBCC042, SBCC073, and SBCC146 - cultivars Cierzo, Orria, and Scarlett.	Spain	automated phenotyping platform	youngest fully expended leaves	▪ Biomass production ▪ Physiological traits ▪ Photosynthesis efficiency	(Boudiar et al., 2020)
Twenty-three barley varieties	Romania	Drought stress induced by polyethylene glycol in Petri dishes	Germination	▪ Germination percentage	(Adriana, 2020)
Eighty genotypes	Worldwide	Drought stress in a greenhouse under controlled conditions	Three‐leaf stage	▪ Biomass production ▪ Relative water content ▪ Stomatal conductance and density ▪ Na^+^, K^+^, and Cl^−^ measurements ▪ Drought tolerance indices	(Hasanuzzaman et al., 2019)
Giza 123-135, and 2000	Egypt	Drought stress induced by polyethylene glycol	Germination, vegetative stages	▪ Germination traits ▪ Biomass production ▪ Physiological and biochemical traits ▪ Molecular traits	(Hellal et al., 2018)
Ten genotypes	Sweden, Iran, ICARDA, CIMMYT, USA, Egypt, Italy	Drought stress under field conditions	Full maturity	▪ Drought tolerance indices	(Arshadi et al., 2018)
12 genotypes	Mediterranean area	Drought stress under field conditions	Full maturity	▪ Morphological traits ▪ Water use efficiency ▪ Drought tolerance indices	(Mansour et al., 2017)
Sensitive (Maresi) and tolerant (Cam/B1) barley genotypes	Poland	Drought stress in pots in greenhouse under controlled conditions	Stem elongation	▪ Chloroplasts form and functioning ▪	(Filek et al., 2015)
Thirty-seven genotypes	Worldwide	Drought stress induced by polyethylene glycol	Germination, vegetative, and reproductive stages	▪ Germination percentage ▪ Biomass production ▪	(Barati et al., 2015)
Georgie, Lubuski, Maresi, and Sebastian, Syrian cultivars Express and Saida, and breeding lines Cam/B1//CI 08887/CI 05761, Harmal02//Esp/1808-4L and M. Dingo/Deir Alla 106,	European genotypes	Drought stress in environmental room under controlled conditions	Tillering stage	▪ Biomass production ▪ Relative water content ▪ Water use efficiency ▪ Net photosynthetic yield ▪ Fv/Fm ratio ▪ Proline and sugar content ▪ Expression profile of genes	(De Mezer et al., 2014)
Yousof and Morocco genotypes	Iran	Drought stress in a greenhouse under controlled conditions	Youngest fully expended leaves	▪ Dry matter and relative water content ▪ Photosynthetic efficiency	(Ghotbi-Ravandi et al., 2014)
Sensitive genotype 004186 and tolerant genotype 004223	Pakistan	Drought stress induced by polyethylene glycol	Seedling development	▪ Proteomic analysis	(Kausar et al., 2013)
Ten genotypes	Iran	Drought stress under field conditions	Full maturity	Drought tolerance indices	(Ajalli and Salehi, 2012)
Sixteen Iranian genotypes	Iran	Water shortage in plastic pots	Flowering period till maturity	Drought tolerance indices	(Nazari and Pakniyat, 2010)
47 Tibet annual wild barley genotypes	China	Drought stress in a hydroponic system	Tillering stage	▪ SPAD value ▪ Shoot and root weights	(Zhao et al., 2010)
12 genotypes	Iran	Drought stress under field conditions	Full maturity	▪ Agronomic traits ▪ Physiological traits	(Vaezi et al., 2010)
Twenty-five autumn barley genotypes (varieties and duble- haploid lines)	Romania	Drought stress under field conditions	Full maturity	▪ Drought tolerance indices	(Velicevici et al., 2010)
Martin, HS41-1, and Moroc9-75	North Africa	Water shortage in plastic pots under controlled conditions	Reproductive stage	▪ Relative chlorophyll content ▪ Ratio Fv/Fm ▪ Grain yield ▪ Differential gene expression	(Guo et al., 2009)

**Figure 2:**
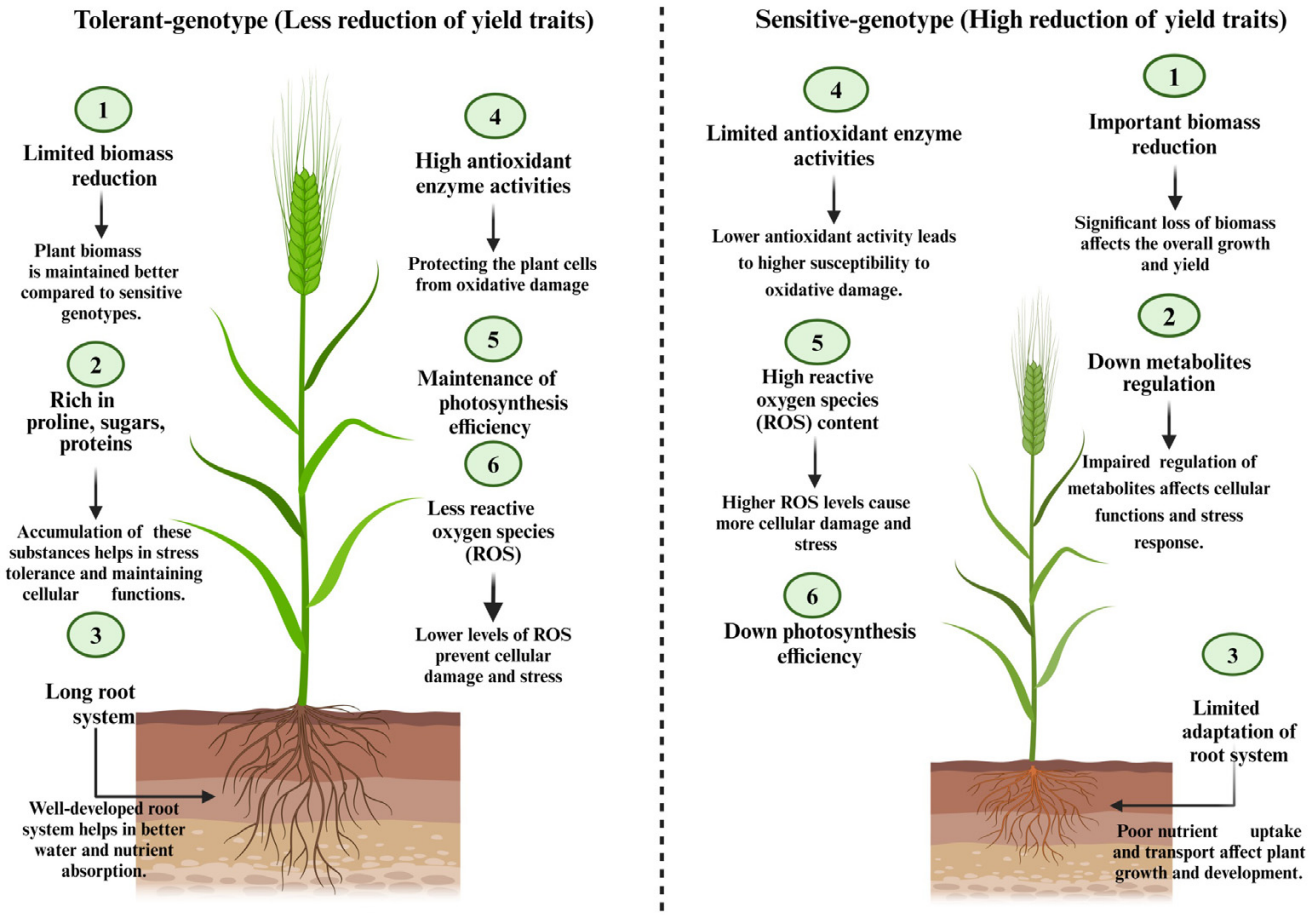
Essential physiological and biochemical differences between tolerant and sensitive barley genotypes.

The selection might be also in the vegetative stage, based on various traits. Selecting drought-tolerant cultivars in this stage is based on morphology, biomass production, physiology, and biochemistry (**Figure 2**).

i). Plant height, root length, root ramification, number of tillers per plant, number of leaves per plant, internode length, flag leaf area, and number of main stem nodes are among the morphological traits frequently investigated in barley plants (Jabbari et al., 2018). In barley as in other cereals, several research studies reported a significant association of these traits to the grain yield trait (Boussakouran et al., 2019), which makes morphological traits effective for barley breeding programs in various growth phenological stages. Here, under drought stress, genotypes that showed less morphology affection are considered more tolerant.

ii). Evaluating the functioning of certain physiological and biochemical traits is widely adopted in breeding studies for the selection of drought tolerant genotypes (Sallam et al., 2019). In this context, the traits evaluated are numerous. In relation with water relations, the relative water content (RWC) index is the most measured trait that reflect the ability of plant to keep cell turgor under stressful condition (Lugojan and Ciulca, 2011), in numerous studies, under drought condition, the trait correlated negatively with grain yield (Samarah, 2005; Gonzalez et al., 2010; Aboughadareh et al., 2013). Genotypes that less reduced RWC traits are considered more drought tolerant (Sallam et al., 2019). Water loss rate shows the balance between transpiration rate and the amount of water supplied to the leaf. Drought tolerant genotypes shows less water loss rate reduction when compared to the sensitive ones (Izanloo et al., 2008). It is widely recorded that drought stress induces strong reduction in plant macro and micro-nutrient uptake (Noman et al., 2018), which affect plant growth and leads to reduces yield production (Sardans et al., 2008). Based on this fundings, many barley breeding studies includes some nutrients uptake in the traits studied (Ayad et al., 2010; Ahmed et al., 2013), the genotypes showed less nutrient uptake reduction under drought stress were considered more tolerant. Every genotypes selection study based on physiological and biochemical traits must investigate the oxidative statue in plant cells (Sallam et al., 2019), which is released by the quantification of reactive oxygen species (ROS) and antioxidants enzyme activities. High ROS content such as H_2_O_2_, superoxide radicals (O_2_), and hydroxyl radicals (OH), resulting stressful environment affects the cellular redox potential which leads to the oxidation of proteins, nucleic acids, chlorophyll pigment, and many other compounds (Hasanuzzaman et al., 2018, 2020), which alters the function of various apparatus and affects plants productivity. When compared to the sensitive ones, drought-tolerant genotypes showed less ROS content increase under drought stress coupled with important increase in antioxidant enzyme activities such as catalase, peroxidases, superoxide dismutase, and reductases (Guan and Scandalios, 2000). The tolerant genotypes showed high ability to increase the activity of antioxidant enzymes in stressful conditions (Guan and Scandalios, 2000). However, this ability is less recorded in sensitive genotypes. ROS accumulation in plant cells induces the peroxidation of membrane lipids (Sharma et al., 2017), which can investigated by measuring malonaldehyde (MDA) content. Low MDA content is widely linked to the stress tolerance (Ferioun et al., 2023a). Under stressful conditions, plants accumulate organic and inorganic osmolytes in cell cytosol such as sugars, proteins, amino-acids, and polyols as a drought tolerance mechanism (Loutfy et al., 2012). Solutes accumulated play many roles especially in osmo-protection, osmotic regulation, ROS neutralization, and macro-molecules protection (Sallam et al., 2019). Under drought stress, proline accumulation in barley leaves is widely reported (Hellal et al., 2018; Ferioun et al., 2023a, b), the roles attributed to this amino-acid are numerous, the most recorded are in relation with osmo-regulation, scavenging free radicals, membranes steadiness (Marcińska et al., 2013). It is important to sign that very limited species and genotypes can accumulates enough proline under stressful conditions (Zali and Ehsanzadeh, 2018) which make this criteria very important to consider it in barley breeding studies. Given the effect of drought stress on photosynthesis traits in barley plants, the evaluation of photosynthesis apparatus becomes highly important in drought-tolerant genotypes selection. Photosynthesis efficiency might be investigated adopting various parameters such as measuring leaves chlorophyll content, photosystem II efficiency (chlorophyll fluorescence), CO_2_ incorporation, and stomatal conductance. Genotypes that can preserve high scores in photosynthesis indices under drought stress might be considered as an important choice for barley breeding program.

At full maturity stage, the evaluation of drought tolerance might be carried out based on agronomic traits such as grain yield (GY), thousand grain weight (TGW), number of grains per spike, spike number per plant, grains per square meter… (Kadam et al., 2014). Furthermore, many breeders use drought tolerance indices based on grain yield under non-stressful and stressful conditions such as susceptibility index (SSI) for the fraction of genotypic productivity (Fischer and Maurer, 1978), mean productivity index (MP) (Mohammadi, 2020), The tolerance index (TOL) (McCaig and Clarke, 1982), stress tolerance index (STI) (G. C. J. Fernandez, 1992), Harmonic mean productivity (HMP) (Jafari et al., 2009), and geometric mean productivity (GMP) (G. C. J. Fernandez, 1992). In this context, many studies in many countries have been carried out to describe the suitable indices for selecting barley drought- tolerant genotypes. The most cited indices were STI, MP, and GMP (Nazari and Pakniyat, 2010; Ajalli and Salehi, 2012; Arshadi et al., 2016; Saed-Moucheshi et al., 2021).

#### 5.1.2 Genetic variation between drought-tolerant and drought-sensitive barley genotypes in response to drought stress

Barley genome sequencing has emerged in recent years, making it easier to understand the molecular mechanisms. Scanning the genetic patterns involved in drought tolerance in barley (*Hordeum vulgare* L.) will facilitate the control of molecular mechanisms of drought tolerance (Karunarathne et al., 2023). Up to date, several studies have been executed in this area, and have revealed different genes and motifs associated with drought resistance/tolerance in barley. Xiong et al. (2023) have compared the drought sensitivity/tolerance of 100 barley genotypes at the seedling period. Those genomes from International Barley Core Selected Collection (BCS), (BCS24 for tolerant genotypes and BCS8 for sensitive ones) have been treated under two planting conditions. Using Genome-Wide Association Study (GWAS) and RNA-Seq methods, this study led to the identification of numerous drought tolerance controlling markers. In this study, 20 SNPs and 41 candidate genes located on chromosomes 2, 5, and 6 have been identified using GWAS. In addition, RNA-seq analysis has confirmed the results of GWAS and revealed more differentially expressed genes in BCS8 and BCS24 (Zhao et al., 2010; Xiong et al., 2023).

Feng et al. (2020); Fu et al. (2022). Gao et al. (2022), and Pan et al. (2022) have revealed new drought-protecting genes, more than 60 genes involved in plant protection against drought stress have been made known thanks to the abovementioned studies. Another study was conducted on a collection of 218 worldwide spring barley genomes, 108 from Europe, 45 from West Asia and North Africa, 36 from East Asia, and 29 from the Americas, this study identified 26 genomic regions localized on chromosomes 1, 2, and 5 considered as the precursors of several candidate genes involved in drought tolerance. Among the 26 revealed genes, 9 adaptive genes are drought-specific or control-specific, and 17 constitutive genes are involved in the genetic variation of the traits studied under both control and induced drought. On chromosome 1, no control-specific genes were identified, while four of them are drought-specific and the remaining 9 are constitutive. The genes located on chromosome 2 are 8 adaptive and 3 constitutive, whereas those identified on chromosome 5 are strictly constitutive. Putative candidate genes in this study, especially those that are drought-specific, are poised to be used for a wider application in crop molecular breeding (Thabet et al., 2018). The majority of executed studies in this area have identified several common drought-controlling genes in barley (**Table 2**), some of those genes have been promisingly used in numerous editing studies trying to improve barley resistance against drought stress (Karunarathne et al., 2023).

**Table 2:** Important genes involved in barley drought tolerance, their location, and functional annotation.

GeneID	Location	Functional annotation	Reference
HORVU6Hr1G067660	Chromosome 6	Phosphoribulokinase / Uridine kinase family	(Fu et al., 2017; Feng et al., 2020, 2022; Geng et al., 2021; Gao et al., 2022; Pan et al., 2022; Karunarathne et al., 2023; Xiong et al., 2023)
HORVU6Hr1G067670		Secretory carrier-associated membrane protein 5	
HORVU6Hr1G067910		Protein NRT1/ PTR FAMILY 8.3	
HORVU6Hr1G067920		Protein NRT1/ PTR FAMILY 8.1	
HORVU6Hr1G067930		DNA topoisomerase 2	
HORVU6Hr1G067680		WRKY DNA-binding protein 35	
HORVU6Hr1G067700		AT-hook motif nuclear-localized protein 20	
HORVU6Hr1G067740		Protein NRT1/ PTR FAMILY 8.3	
HORVU6Hr1G067870		60S ribosomal protein L12–1	
HORVU6Hr1G067880		Guanosine-3′	
HORVU6Hr1G067890		5′ -bis(diphosphate) 3′-pyrophosphohydrolase	
HORVU6Hr1G067980		Protein NRT1/ PTR FAMILY 8.3	
HORVU6Hr1G067760		Protein kinase superfamily protein	
HORVU6Hr1G067840		ADP-ribosylation factor 1	
HORVU5Hr1G055840	Chromosome 5	Ubiquitin-conjugating enzyme family protein	
HORVU5Hr1G055850		sugar transporter 14	
HORVU5Hr1G055260		NBS-LRR type disease resistance protein RPG1-B-like	
HORVU5Hr1G055270		GDP-mannose transporter	
HORVU6Hr1G092630	Chromosome 6	Wound-induced protein	
HORVU6Hr1G092640		Disease resistance protein RPM1	
HORVU6Hr1G092650		Disease resistance protein RPM1	
HORVU6Hr1G092680		Disease resistance RPP8-like protein 3	
HORVU6Hr1G092690		Disease resistance protein RPM1	
HORVU5Hr1G075540	Chromosome 5	Oxidoreductase, zinc-binding dehydrogenase family protein	
HORVU5Hr1G075550		Oxidoreductase, zinc-binding dehydrogenase family protein	
HORVU5Hr1G075560		Myosin heavy chain-related protein	
HORVU5Hr1G075570		FASCICLIN-like arabinogalactan 1	
HORVU5Hr1G075590		unknown function	
HORVU5Hr1G075600		cytidine deaminase 1	
HORVU5Hr1G000750		undescribed protein	
HORVU5Hr1G000760		Pentatricopeptide repeat-containing protein	
HORVU5Hr1G000770		Flavoprotein, HI0933 family	
HORVU5Hr1G000780		H(+)-ATPase 11	
HORVU5Hr1G000800		Protein NRT1/ PTR FAMILY 2.11	
HORVU6Hr1G068060	Chromosome 6	Tudor/PWWP/MBT superfamily protein	
HORVU6Hr1G091800		RING/U-box superfamily protein	
HORVU6Hr1G091840		MATE efflux family protein	
HORVU6Hr1G091850		MATE efflux family protein	
HORVU6Hr1G091860		rRNA/tRNA 2′ -O-methyltransferase fibrillarin-like protein 1	
HORVU2Hr1G111640		Plasma membrane ATPase	
HORVU1Hr1G048400	Chromosome 2	methyltransferase-like protein 17 (mitochondrial)	
HORVU1Hr1G050760	Chromosome 1	Inositol-tetrakisphosphate 1-kinase 1	(Alqudah et al., 2016, 2018; Thabet et al., 2018)
HORVU1Hr1G048410		zinc finger HIT domain-containing protein 2	
HORVU1Hr1G050580		DnaJ homolog subfamily C member 13	
HORVU1Hr1G050650		ARM repeat superfamily protein	
HORVU2Hr1G009970		eukaryotic aspartyl protease family protein	
HORVU2Hr1G010400	Chromosome 2	Chromosome 3B, genomic scaffold	
HORVU2Hr1G023710		SNARE-associated Golgi protein family, soluble N-ethylmaleimide-sensitive factor (NSF) attachment protein receptors	
HORVU2Hr1G023840		rhomboid protein-related	
HORVU2Hr1G023890		myosin-J heavy chain	

### 5.2 Bioinoculants

Microbial inoculants might be constructed using live microorganisms such as bacteria, fungi, and/or algae with important abilities of bio-stimulation and/or bio-protection (Lima et al., 2020; Nayana et al., 2023; Sagar et al., 2024). The application of these biofertilizers might be in the plant rhizosphere, through seed emergence in a biofertilizer solution, or by application to the plant surface (Bittencourt et al., 2023). Many species of these microbes have shown an important ability to improve plants tolerance to drought stress (Asghari et al., 2020; Ilyas et al., 2020; Hamid et al., 2021; Ashry et al., 2022; Mawar et al., 2023), which might be attributed to: (i) enhancing the content of assimilable nutrients via mineral solubilization, N_2_ fixation, organic compounds decomposition, and siderophores production, (ii) the secretion of hormones such as auxins and gibberellins, which ameliorate morphological and physiological status in plants and (iii) secretion of extracellular polymeric substances that helps retain moisture (Ilyas et al., 2020) and their role plant photosynthesis (Uarrota et al., 2022).

The attempts to commercialize bio-inoculants began for the first time in the twentieth century based on nodulating and non-nodulating bacteria and algae (Bittencourt et al., 2023). Ever since, the global adoption of bio-inoculants by farmers has been increasing annually (Santos et al., 2019; Kapadia et al., 2021). Gradually, several studies have described bio-inoculants formulated based on various microbes, demonstrating a remarkable enhancement in the growth of various plants under diverse environmental conditions (Etesami and Maheshwari, 2018; Rebi et al., 2022). In cereals, Rebelo Romão et al. (2022) reported the use of maize seeds encapsulated with xerotolerant microorganisms for the protection of maize plants against water shortage conditions. Kaboosi et al. (2023) reported the ability of *Serendipita indica* to enhance drought tolerance in maize. Bangash et al. (2021) reported the formulation of biofertilizer for improving wheat yield and growth in rainfall farming system.

The studies on bio-inoculants encompass not only the investigation of microbes but also the refinement of formulation techniques. Bio-inoculants formulation involves developing a homogenous combination of a chosen beneficial strain and an appropriate carrier that can stabilize and protect the strain throughout storage and transportation. Effectiveness, non-polluting nature, ease of biodegradation, high water retention capacity, and sufficient shelf life are all desirable qualities in a bioformulation. In literature, several types of formulation are recorded such as: solid, liquid, and polymeric formulations (Chaudhary et al., 2020).

### 5.3 Intercropping

Intercropping, also known as polyculture, refers to the simultaneous cultivation of two or more distinct crop species within a single field throughout a growing season (Elouattassi et al., 2023). It is widely embraced globally as a significant sustainable approach due to its beneficial impact on crop productivity, yield stability, as well as enhanced efficiency in utilizing nutrients and water resources (Fan et al., 2020; Hussain et al., 2023). The benefit of effective water use in intercropping arises due to variations in water needs among intercrops across spatiotemporal differences (Dong et al., 2018). Optimal irrigation amount and timing are crucial in aligning crop water needs with water availability, thereby enhancing the compatibility between the intercropped plants (Yin et al., 2020). Woldeamlak et al. (2006) conducted a drought experiment and discovered that the yields of wheat and barley mixtures remained notably consistent between the drought treatments and controls. These mixtures exhibited higher yields under both seedling and heading stage drought stress conditions. The authors hypothesized that this increased productivity in mixtures might be attributed to the later maturation of wheat, enabling it to utilize soil moisture more efficiently after the barley had matured, leading to a more comprehensive utilization of available moisture compared to the individual crop components. According to Yin et al. (2017), intercropping proves advantageous in elevating photosynthetic resources, such as leaf area index (LAI) and leaf area duration (LAD), while facilitating the transfer of photosynthetic substances from vegetative organs to grains. This process contributes to improving water use efficiency (WUE) by boosting crop yield. Ikram ul Haq et al. (2020) have shown that planting 8 rows of barley on beds alongside Egyptian clover grown in 120 cm irrigation furrows exhibited superior performance and emerged as the most promising approach concerning sustainability, profitability, and efficiency in utilizing irrigation water. Furthermore, a recent study done by (Liang et al., 2023) demonstrated that there was no significant difference in barley grain yield observed between barley monocropping and barley-pulse intercropping, particularly under conditions of limited irrigation. This suggests that barley-pulse intercropping systems might be well-suited for regions facing restricted irrigation resources or engaged in dryland farming. Cereal-pulse intercropping can enhance the use of soil moisture for crop growth and development by complimentary root growth coupled with improved water and nutrient uptake (Stomph et al., 2020; Gowtham et al., 2022; Rafique et al., 2023; Tanveer et al., 2023). Additionally, employing optimized tillage and mulching methods improves the harmony between water requirements for crop growth and the available water supply, thereby enhancing the water use efficiency (WUE) of intercropping (Yin et al., 2017; Khan et al., 2019; Jabborova et al., 2021; Najafi et al., 2021).

### 5.4 Other technologies

In recent years, there has been a remarkable increase in studies on the utilization of nanoparticles, such as TiO_2_, Ag, SiO_2_, and ZnO, to enhance plant growth under various environmental conditions. The mechanism of action of these nanoparticles is still unclear (Ahluwalia et al., 2021). However, it seems that the use of nanoparticles enhance germination, shoot and root growth, relative water content, nutrients uptake, osmotic status, oxidative status, and photosynthesis efficiency in several plants and under various environmental conditions (Arruda et al., 2015). In barley, the only study found in literature, by Moaveni et al. (2011), described a significant increase in yield in barley plants sprayed with Ti[O.sub.2] nanoparticle compared to those non-sprayed under field conditions. On the other hand, the use of these compounds with over dose can affect negatively plants growth. Indeed Martínez-Fernández et al. (2016) reported negative effects of nanoparticles on *Helianthus annuus* L., such us root dehydration and the up regulation of oxidative stress. Moulick et al. (2020), noted a possible long-term negative effect of these compounds on ecosystems.

Biochar, produced by the thermal treatment of biomass (wood, crop residues, or animal manure) under limited oxygen conditions, is widely cited in literature as fertilizer that can promote plants growth and enhance their tolerance against stressful conditions (Haider et al., 2022). In barley, Hafez et al. (2020) reported a considerable beneficial effect of biochar on antioxidant enzymes, anatomic characters, and osmotic status under drought stress. Nasiri et al. (2023) and Gul et al. (2023) recorded a significant increase in barley yield treated with biochar under drought stress. on the other hand, some disadvantages of biochar have been reported, such as soil alkalinization, unavailability (binding) of nutrients in the soil, binding of pesticides and herbicides, accumulation of heavy metals in soils treated with biochar, and affecting rhizosphere microorganisms (Ahluwalia et al., 2021).

Very recently, several studies have reported the utilization of hydrogels, a polymer capable of retaining a significant amount of water and nutrients. This involves the storage of water and nutrients for root plants, which can be utilized during periods of water scarcity (Zhang et al., 2021). However, the use of this technique is still limited in greenhouse setups.

## 6 Plant growth promoting rhizobacteria: An alternative for enhancing *Hordeum vulgare* productivity

**Figure 3:**
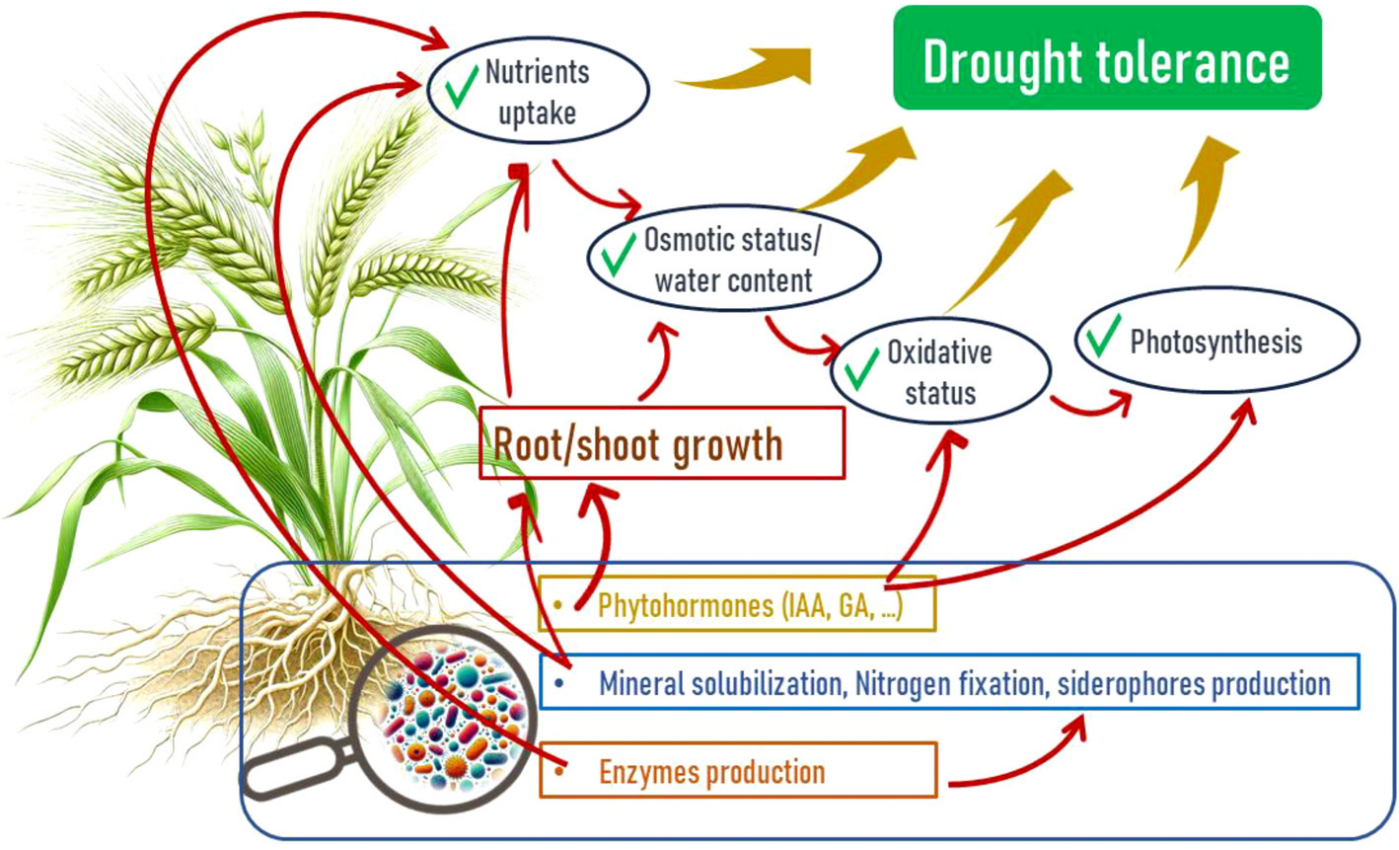
Mechanisms of barley drought tolerance enhancement by Plant Growth Promoting Rhizobacteria (PGPR) through direct and indirect effects.

Of different microbial populations present in the rhizosphere, bacteria are the most abundant microorganisms (Kaymak, 2010), and among these bacteria, we find Plant Growth-Promoting Rhizobacteria (PGPR). These represent a group of beneficial microorganisms inhabiting the rhizosphere and playing a crucial role in enhancing plant growth, yield, and crop quality (**Figure 3**) (Etesami and Maheshwari, 2018) under non-stress and stress conditions through various direct and indirect mechanisms (Mahmood et al., 2014).

Under normal and stressful conditions (abiotic (drought, salt, and heavy metal) and biotic (phytopathogenic species), PGPRs can affect plants directly by facilitating the uptake of certain nutrients by the plant through the synthesis of certain compounds by the bacteria and indirectly by reducing or preventing the undesirable effects of one or more phytopathogenic organisms (Kumar et al., 2019).

### 6.1 Direct mechanisms

Direct promotion is achieved through several mechanisms, including the production of phytohormones such as gibberellins, auxins (indole acetic acid), and cytokinin, which play an important role in cell elongation, cell division, tissue differentiation, and apical dominance (Kaymak, 2010). For example, the treatment of two selections of roses (*Rosa canina* and *Rosa dumalis*) and *Pistacia vera* with indole-3-butyric acid (IBA) improved the rooting rate and increased the number of lateral roots of the plants (Ercisli et al., 2004; Orhan et al., 2007).

Other direct mechanisms include nitrogen fixation and the solubilization of certain nutrients that limit plant growth. Physiological fixation of nitrogen by rhizobacteria makes it more accessible to plants, and can be symbiotic, forming nodules at plant roots, or non-symbiotic (Bellés-Sancho et al., 2023). To assimilate nitrogen, certain bacteria such as *Azotobacter* spp. and *Azospirillum lipoferum* have the capacity to synthesize the enzyme nitrogenase (Niewiadomska et al., 2018). Inoculation of *Trifolium repens* with Rhizobium and PGPR showed an increase in nodules, nitrogenase levels, and nitrogen content in roots and shoots compared with the control (Matse et al., 2020).

In the soil, phosphorus exists in two forms: inorganic and organic. Because of its low solubility, micro-organisms play a crucial role in the process of dissolving and mineralizing phosphorus, notably through the production of organic acids and phosphatases that catalyse the hydrolysis of phosphoric acid esters (Alori et al., 2017). In other words, phosphate solubilization is achieved through acidification, chelation, exchange reactions, and gluconic acid production (Hameeda et al., 2008). Phosphate solubilizing bacteria (PSB) are omnipresent (Gyaneshwar et al., 2002), where *Bacillus*, *Enterobacter*, *Erwinia* and *Pseudomonas* are among the most potent genera (Podile and Kishore, 2006). *Mesorhizobium mediterraneum* was able to effectively mobilize tricalcium phosphate and insoluble phosphates added to the soil and increase the P, N, K, Ca, Mg and dry matter content of barley and chickpea (Peix et al., 2001).

### 6.2 Indirect mechanisms

The indirect mechanisms comprise suppression of phytopathogens by the production of volatile HCN, siderophores, volatile metabolites, and ammonia, etc., induced systemic resistance in the host plant and competition with the pathogen for space and nutrients (Etesami and Adl, 2020).

The trivalent form of iron hydroxide (Fe^3+^) in the soil is difficult for plants to absorb, and to facilitate this uptake, bacteria produce low-molecular-weight molecules called siderophores. These siderophores act as iron chelators while making iron accessible to plants in case of deficiency (especially in neutral and alkaline soils) (Khan et al., 2018). Siderophores not only chelate iron but can also form stable complexes in the presence of other metals (Gu et al., 2020). Siderophores play a crucial role in plant disease management by depriving pathogens of iron, leading to their inhibition. For example, siderophores secreted by *Pseudomonas* are the main factors inhibiting the growth and development of fungal pathogens, including *Colletotrichum dematium*, *Rhizoctonia solani* and *Sclerotium rolfsii* (Sharma and Johri, 2003).

Volatile organic compounds (VOCs) secreted by rhizobacteria are low molecular weight compounds capable of diffusing through different matrices, including biological membranes, water, soil and air (Lemfack et al., 2014; Etminani et al., 2024) to provide inter- and intra-organism communication (Farag et al., 2013; Mhlongo et al., 2018). They play a crucial role in improving plant growth by modulating levels of phytohormones such as ethylene, auxin, and jasmonic acid and tolerance to abiotic stress (Song et al., 2008). In addition, a multitude of VOCs (benzothiazole, cyclohexanol, n-decanal, dimethyl trisulfide, 2-ethyl 1-hexanol, and nonanal) produced by bacteria in the rhizospheres of canola and soybean inhibit sclerotia and ascospore germination and mycelial growth of *Sclerotinia sclerotiorum* under both laboratory and field conditions (Fernando et al., 2005).

Hydrogen cyanide (HCN) is a toxic secondary metabolite that inhibits aerobic respiration activities (Williams et al., 2006). It is produced only in a few bacterial species, including *P. aeruginosa* (Neerincx et al., 2015). HCN produced by rhizobacteria plays an important role in the biological control of phytopathogens and pests (Suryadi et al., 2019). For example, HCN synthesised by *Pseudomonas* sp. inhibits certain pathogenic fungi (Suryadi et al., 2019), and that synthesised by *P. chlororaphis* O6 has shown nematocidal activity (Kang et al., 2018). Ammonia (NH_3_^+^) production helps to satisfy the nitrogen demand of host plants and in excess reduces plant colonization by pathogens. This NH_3_^+^ production is achieved by the hydrolysis of urea by nitrogenase into ammonia and carbon dioxide (Mbai et al., 2013), degradation of plant ACC, or deamination of amino acids (Etesami and Adl, 2020).

In 1990, a discovery revealed that certain non-pathogenic bacteria could prevent the metabolic changes caused by pathogen attacks by triggering a systemic response in the plant called induced systemic resistance (ISR) (Ali et al., 2022; Desai et al., 2023; Gowtham et al., 2024). This response was initially discovered in the plant model *Arabidopsis thaliana* and has since been observed in many plant species. In *A. thaliana*, the ISR response is mediated by ethylene and jasmonic acid as signal transducers. Additionally, the NPR1 protein is involved, which induces the expression of other proteins different from PRs. For example, *P. fluorescens* strain WCS417 was shown to be effective against *Fusarium oxysporum* f. sp. *dianthi* on carnations, acting protectively even when the bacteria remained confined to the root system of the plant (van Peer, 1991).

This phenomenon was observed with several PGPR strains applied to cucumber roots, providing protection against the anthracnose fungus *Colletotrichum orbiculare* during subsequent inoculations (Wei et al., 1991).

Through these mechanisms, the treatment of plants in general and barley specifically with PGPRs affects growth parameters positively and offers protection against abiotic and biotic stresses (**Table 3**).

**Table 3:** Studies for the enhancement of barley drought tolerance employing bioinoculant based on plant growth promoting rhizobacteria (PGPR).

Bacteria	PGP traits of bacteria	Effects on barley plants	Treatments	References
*Pantoea agglomerans* *Streptomyces swartbergensis* *Pseudomonas zanjanensis* *Streptomyces cahuitamycinicus* *Ensifer meliloti*	Phosphate and potassium solubilization Auxin and siderophore production Nitrogen fixation ACC deaminase secretion	Enhancement of plant biomass, photosynthesis efficiency, and biochemical traits.	Full irrigation regime Drought stress	(Slimani et al., 2023b)
PGPR consortia	–	Enhancement of physiological and biochemical traits	Full irrigation regime Drought stress	(Slimani et al., 2023a)
*Providencia rettgeri*	IAA, EPS, NH_3_, pectinase, chitinase, phosphatase acid and phosphatase alkaline production, resistance to water stress, nitrogen fixation and P solubilization	Improvement of shoot dry weight, relative water content, chlorophyll pigments content and photosynthesis efficiency Decrease in electrolytes leakage, MDA and hydrogen peroxide contents	Full irrigation regime Drought stress	(Ferioun et al., 2023b)
*Serratia odorifera*	–	Enhancement of plant biomass production, chlorophyll content, and antioxidant enzyme activities	Full irrigation regime Drought stress	(Gul et al., 2023)
*Bacillus subtilis*	–	Improvement of plant growth and biomass, photosynthetic efficiency, antioxidant enzymes and mineral absorption, - Reduction of exudation of organic acids and oxidative stress indicators in roots	No stress Metal stress by chromium	(Zhu et al., 2023)
*Bacillus mycoides*	–	Enhancement of shoot and root lengths and weight, leave traits, chlorophyll pigments content, and photosynthesis efficiency Reduction of MDA, H_2_O_2_, EL and organic acids	None-stressful conditions Metal stress by Cadmium	(Ma et al., 2023)
*Pantoea* sp & *Pseudomonas* sp	–	Enhancement of leaf respiration and transpiration - Photoinhibition, and the risk of oxidative stress	Full irrigation regime Drought stress	(Abideen et al., 2022)
*Bacillus mojavensis* *Pseudomonas fluorescens*	Resistance to salt stress and production of IAA and proline.	Increase of shoot and root dry weights Enhancement of stomatal conductance and CO_2_ assimilation Decrease of root and shoot Na^+^ concentrations Improvement of leaf water potential.	None-stressful conditions Salt stress	(Mahmoud et al., 2020)
Pseudomonas fluorescens Pseudomonas putida	*-*	Enhancement of root weights, chlorophyll content, and relative water content Enhancement of abscisic acid, saliciylic acid and indole acetic acid biosynthesis. up-regulation of the genes of jasmonic acids and ethylene biosynthesis. Enhancement of the expression of nitrate transporter and antioxidant genes.	Salt stress	(Zaib et al., 2020)
*Pantoea agglomerans*	Inorganic and organic P solubilization K mobilization siderophores, ACC deaminase and IAA production. Nitrogen fixation Tolerance to salinity, cold and drought stresses.	Increase of plant height and weight, chlorophyll content, Enhancement of mineral nutrition (Mg and K) Induction of resistance against the fungal pathogen	None-stressful conditions Nutrients deficit	(Rahman et al., 2018)
*Azotobacter* (strain12), *Pseudomonas* (strainp-169), *Azospirillum* (strain OF)	–	Enhancement of grain yield, biological Yield, and thousand grain weight	Full irrigation regime Drought stress	(Rezaei et al., 2017)
*Pseudomonas putida*		Enhancement of ions absorption Increase of the cell membrane stability, photosynthesis rate and biomass measurements.	- Salt stress	(Jodeh et al., 2015)
*Hartmannibacter diazotrophicus*	ACC-deaminase activity	Improvement of plant growth, and water content Decrease of sodium uptake and the ethylene emission.	None-stressful conditions Salt stress	(Suarez et al., 2015)
*Curtobacterium flaccumfaciens*	IAA production and Phosphate mobilization	Increase in germination rate of seeds, and biomass and resistance of leaves, stems and roots to salt stress. *E. garamanticus* aids in the accumulation of water in plant roots	None-stressful conditions Salt stress	(Cardinale et al., 2015)
*Ensifer garamanticus*	IAA production, nitrogen fixation, Phosphate and phytate mobilization	Improvement of plant growth, and water content Decrease of sodium uptake and the ethylene emission.	None-stressful conditions Salt stress	(Suarez et al., 2015)
Strains name not indicated	–	Under drought stress, PGPR enhanced cell membrane stability Increase in root and shoot weight, grain weight per spike, and grain yield	Full irrigation regime Drought stress Full irrigation in greenhouse Rainfall in field	(Rezaei and Pazoki, 2015) (Cakmakci et al., 2014)
*Bacillus* OSU-142 *Bacillus megaterium* M3 *Azospirillum brasilense* Sp.245 *Bacillus megaterium* RC07 *Paenibacillus polymyxa* RC05 *Bacillus licheniformis* RC08				
*Luteibacter rhizovicinus*	Inorganic Phosphate solubilization Siderophores and IAA production	Significant increase in the weight of the aerial part, and the weight and length of the roots.	None-stressful conditions	(Guglielmetti et al., 2013)
*Bacillus megaterium* M3, *Bacillus subtilis* OSU142, *Azospirillum brasilense* Sp245, and *Raoultella terrigena*	–	Enhancement stomatal conductance, leaves water content, and membrane stability.	Full irrigation regime	(Turan et al., 2012)
*Bacillus* RC01*, Bacillus* RC02, *Bacillus* RC03 and *Bacillus* M-13	Nitrogen fixation Phosphate solubilization Amylase positive	Increase in root weight, shoot weight and total biomass weight.	None-stressful conditions	(Canbolat et al., 2006)

### 6.3 Root and shoot growth

The root system is the first part of the plant that is exposed to the PGPR influence. Generally, roots growth and architecture are affected by phytohormones such as auxins, gibberellin, and abscisic acid. The drought stress reduces barley plants length and biomass by affecting phytohormones balance (Barati et al., 2015; Bardehji et al., 2023). The bioinoculant based on PGPR showed a significant increase in various root traits in barley plant grown under drought stress. Ferioun et al. (2023b) described a significant increased effect of *Providencia rettgeri* on RDW. Slimani et al. (2023a) described four PGPR isolates that increased significantly roots length of barley plants under drought stress. In both last studies, PGPR strains were characterized as being able to produce high levels of indole-3-acetic acid (IAA) phytohormone. This last is widely reported in literature in the regulation of cells and tissues plant growth (Etesami and Adl, 2020). The majority of PGPR strains isolated were IAA producer, and many researches in other crops reported a significant relationship between phytohormones secretion and yield production (Ahluwalia et al., 2021) (**Figure 1**).

Several studies described a significant increase in shoot weight and length on barley plants inoculated with PGPR strains when compared to the uninoculated ones (Gul et al., 2023; Slimani et al., 2023a; Ferioun et al., 2023b). The last references described a higher significant positive correlation between shoot growth traits and root ones, which prove that shoot growth is linked to root development induced by PGPRs inoculation. Furthermore, the ability of PGPRs to fixate N_2_ and to solubilize some nutrients such as potassium and phosphate increases nutrient content in plants rhizosphere (Dhaked et al., 2022; Tirry et al., 2023). The increase in water and nutrients assimilation due to the root growth enhancement and nutrients assimilation lead to the improvement of photosynthesis efficiency and biomass production (Raklami et al., 2021). Moreover, the increase in shoot growth might be linked to the enhancement of ACC deaminase activity by PGPRs which promote physiological tolerance of plants under stressful conditions (Danish et al., 2020). This enzyme catalyzes the reaction of 1-aminocyclopropane-1-carboxylic acid (ACC) cleavage into ammonia and α-ketobutyrate, thereby inhibiting the formation of the stress hormone ethylene (Sagar et al., 2020; Naing et al., 2021). In barley, as in other cereals, the inoculation with ACC deaminase PGPR producer increased plant tolerance to drought stress (Chandra et al., 2019; Danish et al., 2020; Slimani et al., 2023b).

### 6.4 Water and osmotic status

Relative water content (RWC) is utilized in several studies as an indicator of plant water status. Generally, RWC is associated with the water taken by leaf tissue and the water lost through transpiration (Lugojan and Ciulca, 2011). High RWC value under stressful conditions reflects a strong ability of plant tissues to maintain their cell turgor pressure under stresses (Ferioun et al., 2023a). The inoculation of barley plants with PGPRs under drought stress increased RWC% when compared to the stressed uninoculated plants (Abideen et al., 2022; Ferioun et al., 2023b); aiding barley plants in mitigating damages associated with decreased cell turgor pressure. This effect is frequently linked to the effect of PGPRs on the opening/closure of stomates, root water absorption, and hydraulic conductivity (Ahluwalia et al., 2021).

Plant cells adjust their osmotic pressure to mitigate the damages caused by fluctuations in osmotic pressure (Sallam et al., 2019). The osmotic adjustment might be released by osmoregulation through solutes accumulation which leads to reduced osmotic pressure in plant cells (Ahluwalia et al., 2021). On the other hand, the osmo-protection might be involved by antioxidant system or by the accumulation of osmo-protectants such as polyamines, sugars, and proline (Zulfiqar et al., 2020; Eswaran et al., 2024). Askarnejad et al. (2021); Slimani et al. (2023a); Ferioun et al. (2023b), and Slimani et al. (2023b) reported a significant impact of PGPR inoculation on sugars and proline contents in barley plants under drought stress, which confirm the strong effect of PGPRs on the adjustment of osmotic pressure and on the enhancement of physiological tolerance.

### 6.5 Oxidative status

In barley plants inoculated with PGPRs, the contents of H_2_O_2_ and MDA under various environmental conditions is significantly fewer than uninoculated plants under drought stress (Zaib et al., 2020; Abideen et al., 2022; Slimani et al., 2023a; Ferioun et al., 2023b). The same references indicated a significant increase in antioxidant (scavenging) enzyme activities (catalase, peroxidase, superoxide dismutase) in barley plants inoculated with PGPRs when compared to the uninoculated ones. Indeed, several studies reported a negative correlation between antioxidant enzyme activities and H_2_O_2_ content (Zhanassova et al., 2021), which confirms that PGPRs reduce oxidative stress induced by water shortage via the up-regulation of scavenging enzyme activities.

### 6.6 Photosynthesis efficiency

In barley, under non-stressful conditions, under drought stress, and in the case of other abiotic stresses, PGPRs enhance photosynthesis efficiency, which improves biomass and organic matter production (Zaib et al., 2020; Ahluwalia et al., 2021; Abideen et al., 2022; Abbasi et al., 2023). PGPRs promote chlorophyll pigments synthesis pathways directly via hormones synthesis or indirectly via the enhancement of water and nutrients uptake (Ghanbarzadeh et al., 2020). This increase in chlorophyll content leads to the optimization of photosynthetic rate and photosystem II efficiency, and the functioning of the complex pigment-proteins (Abideen et al., 2022). This last study reported that, the inoculation of barley plants with *Pantoea* sp. & *Pseudomonas* sp. under drought stress optimized leaf transpiration, gas exchange, as well as stomatal conductance.

## 7 Prospects and challenges for the future

In the next few years, the need for increased outputs, better crop productivity, soil health, and ecologically sound farming is growing. Breeding genotypes resilient to drought has great potential to increase barley's ability to withstand water shortage in future years, but it also comes with a number of difficulties. Through the use of genome editing, genomic selection, and marker-assisted selection (MAS), drought-tolerant barley genotypes may be developed more quickly than ever before as a result of developments in molecular genetics, genomics, and phenotyping technology (Sallam et al., 2019). By using these methods, breeders may quickly accelerate the genetic development of drought tolerance characteristics by identifying and introducing advantageous alleles linked to drought tolerance from broad germplasm pools and wild barley relatives into top breeding lines. On the other hand, there are still a number of difficulties in selecting barley drought-tolerant genotypes despite these technical developments. Drought tolerance is a complex feature involving many interacting physiological, biochemical, and molecular systems, it is difficult to properly deconstruct and regulate these qualities. Moreover, Genotypes x Environment (G x E) interactions and polygenicity are common in the genetic architecture of drought tolerance traits, making multi-environment trials and strong statistical techniques necessary for precise trait assessment and selection. Furthermore, socioeconomic and legal obstacles, such as those pertaining to intellectual property rights, public acceptability, and farmer adoption, stand in the way of the broad distribution and adoption of drought-tolerant barley cultivars.

The strategic use of bio-inoculants to improve barley stress tolerance to drought has great potential in the upcoming years. PGPR bio-inoculants offer a sustainable and agroecological solution (Abbasi et al., 2023). The combination of these bio-inoculants with suitable breeding approach seems promising for developing barley genotypes with enhanced drought resilience indices. To fully realize the promise of bio-inoculants for improving barley's resistance to drought stress, several challenges must be overcome: (i) Field experiments and on-farm demonstrations are necessary to further investigate the efficacy and consistency of bio-inoculant performance under various environmental circumstances and agricultural techniques; (ii) Strong screening and selection processes that take into account genotype-microbiome interactions and compatibility with other agricultural inputs are necessary for the selection and optimization of appropriate bio-inoculant strains for particular barley cultivars and agroecosystems; (iii) It is necessary to address the flexibility, cost-effectiveness, and commercial feasibility of bio-inoculant manufacturing and application technologies in order to enable farmers and stakeholders to use them widely. To overcome these challenges and ensure food security in the face of water scarcity and climate change, interdisciplinary research collaborations, technological advancements, and policy support will be necessary to support the sustainable integration of bio-inoculants into barley cultivation systems.

## 8 Conclusion

Drought strongly influences barley plant physiology and productivity and lowers the economic incomes of barley crops. Genotype breeding is among the solutions widely studied to obtain robust and resilient barley genotypes, aiming to mitigate barley losses caused by water scarcity. Tolerant genotypes exhibit fewer physiological disturbances and maintain relatively high productivity even under stressful conditions. At the same time, bio-inoculants based on PGPRs are considered effective agroecological and sustainable solution. PGPRs used for bio-inoculant construction exhibit strong PGP traits including enhancing nutrients availability, phytohormones secretion, EPS production, and the stimulation of plant systemic defence mechanisms. Barley plants inoculated with these bio-inoculants showed higher physiological resistance and yield productivity. Based on the genotype x microbiome interaction, developing appropriate bio-inoculants for specific barley cultivars and agroecosystems is a highly effective solution that can maximize barley yield production under diverse conditions.
